# Whole genome sequencing data for two individuals of Pakistani descent

**DOI:** 10.1038/sdata.2018.174

**Published:** 2018-09-11

**Authors:** Shahid Y. Khan, Firoz Kabir, Oussama M’Hamdi, Xiaodong Jiao, Muhammad Asif Naeem, Shaheen N. Khan, Sheikh Riazuddin, J. Fielding Hejtmancik, S. Amer Riazuddin

**Affiliations:** 1 The Wilmer Eye Institute, Johns Hopkins University School of Medicine, Baltimore, MD 21287, USA; 2 Ophthalmic Genetics and Visual Function Branch, National Eye Institute, National Institutes of Health, Bethesda, MD 20892, USA; 3 National Centre of Excellence in Molecular Biology, University of the Punjab, Lahore 53700, Pakistan

**Keywords:** DNA sequencing, Genomics, Genetic variation

## Abstract

Here we report next-generation based whole genome sequencing of two individuals (H1 and H2) from a family of Pakistani descent. The genomic DNA was used to prepare paired-end libraries for whole-genome sequencing. Deep sequencing yielded 706.49 and 778.12 million mapped reads corresponding to 70.64 and 77.81 Gb sequence data and 23× and 25× average coverage for H1 and H2, respectively. Notably, a total of 448,544 and 470,683 novel variants, not present in the single nucleotide polymorphism database (dbSNP), were identified in H1 and H2, respectively. Comparative analysis identified 2,415,852 variants common in both genomes including 240,181 variants absent in the dbSNP. Principal component analysis linked the ancestry of both genomes with South Asian populations. In conclusion, we report whole genome sequences of two individuals from a family of Pakistani descent.

## Background & Summary

The Human Genome Project was launched in the early 90s with the goal of establishing a comprehensive sequence of the human genome. The first draft of the human genome was completed in the year 2000 and the complete sequence of the human genome was published a year later^1^. In the following years, research efforts to determine variations in the human genome led to the development of a database of the common variants identified in the human genome^2^.

Advancements in next-generation sequencing (NGS) technologies have driven the development of comprehensive databases of genetic variants from different ethnic populations^3–9^. The 1000 Genomes Project reports human genetic variation profiles from 26 ethnic populations, including one Pakistani (Punjabi), two Indian (Gujarati and Telugu), one Bangladeshi (Bengali), and one Sri Lankan (Tamil) population—all descendants of the Indian subcontinent^9^. Additionally, independent groups have recently published two Indian and two Pakistani genomes with an overall 25×–30× sequencing coverage^10–13^.

## Methods

### Sample collection

The protocol for this study was approved by the Institutional Review Board (IRB) of Johns Hopkins University School of Medicine (Baltimore, MD), National Eye Institute (Bethesda, MD), and National Centre of Excellence in Molecular Biology (Lahore, Pakistan). Both participating members provided informed written consent consistent with the tenets of the Declaration of Helsinki. A small aliquot (~10 ml) of blood sample was collected from each individual and genomic DNA was extracted as previously described^14^.

### Library preparation and next-generation sequencing

Whole genome sequencing (WGS) was performed commercially at the Johns Hopkins GRCF High Throughput Sequencing Center. In brief, 1.0 μg of genomic DNA (gDNA) was fragmented using an E210 Focused-ultrasonicator (Covaris, Woburn, MA) and the quality of gDNA fragmentation was examined using an Agilent 2100 Bioanalyzer (Agilent Inc., Santa Clara, CA). The sheared gDNA was used to prepare paired-end libraries with the TruSeq DNA PCR-Free Library Preparation Kit with an average insert size of 450 bp for both samples (H1 and H2) according to the manufacturer's instructions (Illumina Inc., San Diego, CA). Each library was sequenced in two independent lanes of HiSeq 2500 in paired-end fashion (2×100 bp; Illumina Inc.). The base calls were assigned through Illumina Real Time Analysis software (Ver. 1.17.20) and binary base call (BCL) files were converted to flat file format (qseq.txt) using Illumina BCL Converter software (Ver. 1.9.4). Qseq.txt files were demultiplexed to single-sample FASTQ files using demultiplexer software part of CIDRSeqSuite (Ver. 7.1.0; unpublished).

### Bioinformatics analysis

Lasergene Genomics Suite (DNASTAR, Madison, WI) was used for reference-guided genome alignment. Paired-end raw reads of H1 and H2 were aligned to the human reference genome (GRCh38.p2) using SeqMan NGen (Ver.12; DNASTAR) with default parameters. The mapped reads in BAM file format were subjected to ArrayStar (Ver.12; DNASTAR) for annotation and identification of single-nucleotide variants (SNVs) and indels in both genomes. SNVs and indels were subjected to Ensembl Variant Effect Predictor (VEP) tool to categorize variants as synonymous, non-synonymous, benign or damaging.

### Ancestry Prediction

Principal component analysis (PCA) was performed to examine the ancestral roots of H1 and H2 genomes using algorithms of Peddy (Ver. 0.3.5)^15^. The study utilized the high-performance computational capabilities of the Biowulf Linux cluster at the National Institutes of Health, Bethesda, MD. PCA plots were created using SNPs genotype information obtained from VCF (variant call format) files (from WGS data of H1 and H2) and comparing it with combined ethnic populations from the 1000 Genomes dataset.

## Data Records

All sequencing raw reads for both genomes (H1 and H2) have been deposited in the NCBI Sequence Read Archive (Data Citation 1).

### Data record 1

Chromosomal distribution of total variants in H1 genome (Data Citation 2).

### Data record 2

Summary of functionally annotated variants in H1 genome (Data Citation 2).

### Data record 3

Chromosomal distribution of total variants in H2 genome (Data Citation 2).

### Data record 4

Summary of functionally annotated variants in H2 genome (Data Citation 2).

## Technical Validation

A total of 760,621,746 and 835,451,324 million reads were generated for H1 and H2, respectively (Table 1 and Data Citation 1). The reads were mapped to the human reference genome (GRCh38.p2), resulting in an average coverage of 23× for H1 and 25× for H2 (Table 1). Quality control examination of the sequencing reads revealed that >99% of the sequencing data yielded a PHRED score of 30 or above (PHRED score of 30 represents the probability of 0.001 that the base call is wrong). We did not identify any over-represented adapter sequences in the sequencing libraries.

**Figure 1:**
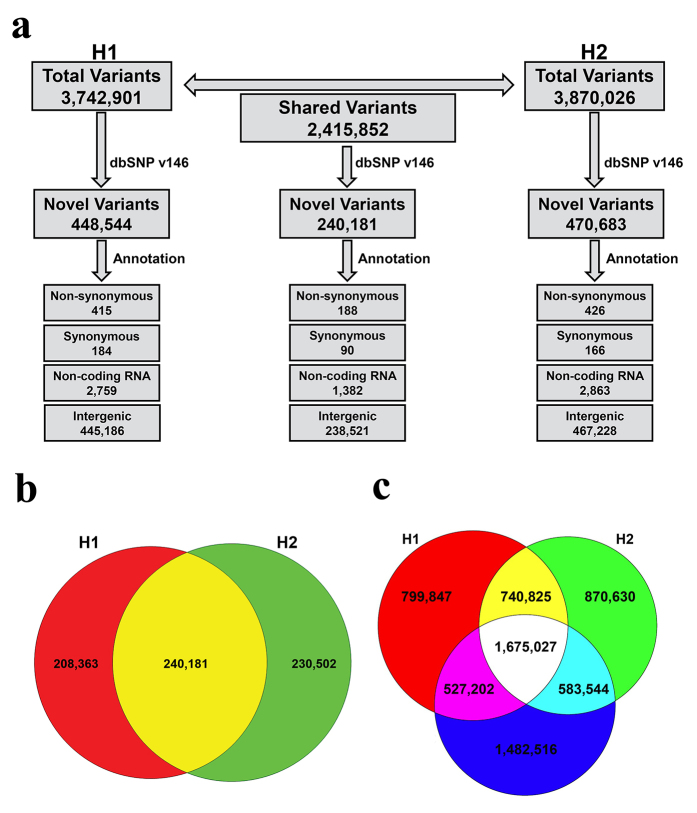
Identification and annotation of variants in two individuals (H1 and H2) from a family of Pakistani descent. (**a**) Flowchart representation of common variants present in H1 and H2 genomes. (**b**) Venn diagram illustrating a comparative analysis of the novel variants in H1 and H2 genomes. Note: Red and green represent novel variants present in H1 and H2, respectively whereas their intersection (yellow) represents novel variants common in both genomes. (**c**) Venn diagram illustrating a comparative analysis of variants identified in H1, H2, and the Caucasian reference genome. Note: Red and green represent variants in H1 and H2 genomes, respectively while the blue circle represents variants in the Caucasian reference genome. The intersections represent variants common in these genomes i.e. yellow: common in H1 and H2; purple: common in H1 and Caucasian reference; light blue: common in H2 and the Caucasian reference; and white: common in H1, H2, and Caucasian reference.

Our analysis identified 3,742,901 total variants in H1 genome including 3,419,449 SNPs and 323,452 indels (Fig. 1a and Chromosomal distribution of total variants in H1 genome, Data Citation 2). Of the 3,419,449 SNPs identified, 1,754,648 were heterozygous while 1,664,801 were homozygous. Annotation of the SNPs identified in H1 genome revealed that 448,544 (12% of the total variants) had not previously been reported (Fig. 1a and Chromosomal distribution of total variants in H1 genome, Data Citation 2). Among these novel SNVs, a small fraction (599) reside within coding DNA sequence (CDS) regions, including 184 synonymous and 415 non-synonymous variants (Fig. 1a and Summary of functionally annotated variants in H1 genome, Data Citation 2). A majority of the SNVs were found in non-coding RNA (2,759) and intergenic (445,186) portions of the genome (Fig. 1a and Chromosomal distribution of total variants in H1 genome, Data Citation 2). A total of 323,452 indels (fewer than ±20 bp) were identified, including 210,802 heterozygous and 112,650 homozygous indels (Chromosomal distribution of total variants in H1 genome, Data Citation 2 and Summary of functionally annotated variants in H1 genome, Data Citation 2). Subsequent analysis revealed 83,747 novel indels, representing 2.23% of the total variants identified in H1 genome. A small fraction (78) of these indels were present in the coding regions while the majority of them (82,549) were present in the intergenic and non-coding RNA (1,120) portions of the genome (Chromosomal distribution of total variants in H1 genome, Data Citation 2 and Summary of functionally annotated variants in H1 genome, Data Citation 2).

In parallel, we identified 3,870,026 total variants in H2 genome, including 3,525,499 SNPs and 344,527 indels (Fig. 1a and Chromosomal distribution of total variants in H2 genome, Data Citation 2). Of the 3,525,499 SNPs identified, 1,831,449 were heterozygous while 1,694,050 were homozygous. The SNPs were annotated against dbSNP that identified 470,683 novel SNVs (12% of the total variants) in H2 genome (Fig. 1a and Chromosomal distribution of total variants in H2 genome, Data Citation 2). Among these novel SNVs, a small fraction (592) were found in CDS regions, including 166 synonymous and 426 non-synonymous variants (Fig. 1a and Summary of functionally annotated variants in H2 genome, Data Citation 2). A majority of the SNVs were localized to intergenic (467,228) and non-coding RNA (2,863) portions of the genome (Fig. 1a and Chromosomal distribution of total variants in H2 genome, Data Citation 2). A total of 344,527 indels (fewer than ±20 bp), including 230,854 heterozygous and 113,673 homozygous indels were identified (Chromosomal distribution of total variants in H2 genome, Data Citation 2 and Summary of functionally annotated variants in H2 genome, Data Citation 2). Subsequent analysis identified 89,517 novel indels, representing 2.31% of the total variants identified in H2 genome. A small fraction (88) of these novel indels were present in coding regions while the majority of them (88,259) were localized to intergenic and non-coding RNA (1,170) portions of the genome (Chromosomal distribution of the total variants in H2 genome, Data Citation 2 and Summary of functionally annotated variants in H2 genome, Data Citation 2).

A comparative analysis identified a total of 2,415,852 variants common in both (H1 and H2) genomes representing ~62% of the total variants identified in each genome (Fig. 1a). Annotation of these common variants identified 240,181 novel variants representing ~6% of the total variants identified in each genome (Fig. 1a,b). In total, we identified 278 common variants in CDS regions including 188 non-synonymous and 90 synonymous variants. We also identified 238,521 common variants in intergenic portions and 1,382 variants in non-coding RNA sequences (Fig. 1a).

In parallel to above, we performed a comparative analysis comparing both (H1 and H2) genomes and a Caucasian reference genome. The Caucasian reference (NA12878) whole genome sequencing raw reads (FASTQ) were downloaded from Sequence Read Archive (SRA) under access number SRX1100298. We identified 740,825 variants common in both genomes but absent from the Caucasian reference genome (Fig. 1c). Of these, 154,930 variants have not been reported previously.

Finally, we employed PCA to predict the ancestral roots of two males (H1 and H2) from Punjabi ethnic group of Pakistan. SNPs genotype of H1 and H2 were compared with combined population datasets of 1000 Genomes project for ancestry prediction. Pakistan is located at the junction of central and south Asia harboring an ethnically diverse population. Among these ethnical populations, Punjabi is the largest ethnicity representing an admixture of highly diverse genetic pool attributed, in part, to the invasions and the settlement of Aryans, Persians, Greeks, Arabs, Turks, Afghans, and Mongols.

**Figure 2:**
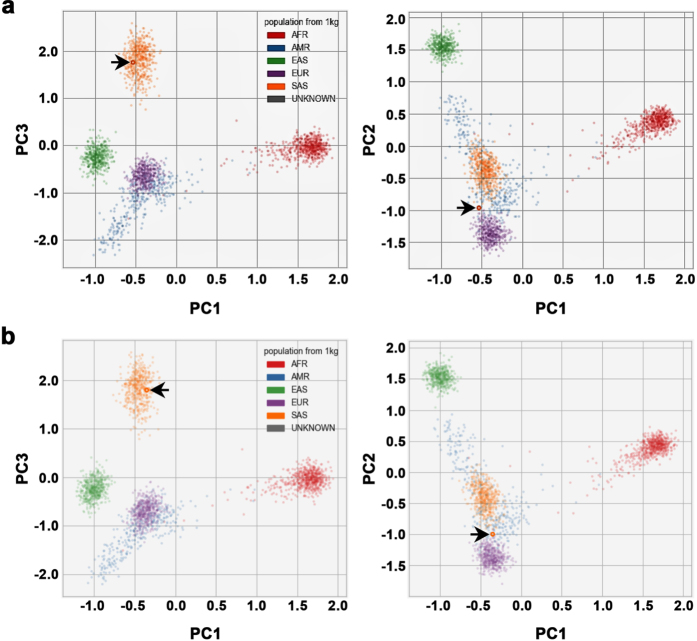
Principal component analysis (PCA) examining the ancestral roots of H1 and H2. (**a**) PCA plots of H1 and (**b**) PCA plot of H2. PCA localized both H1 and H2 (arrows pointing to samples shown as red circles in the PCA plots) with South Asian (SAS) populations in principal component 1 and 3 (PC1 and PC3) and between SAS, and with European (EUR) populations in principal component 2 (PC2). Note: The x-axis represents PC1 while the y- and z-axis represents PC2 and PC3, respectively. African: AFR; Ad Mixed American: AMR; and East Asian: EAS.

PCA localized both H1 and H2 (arrows pointing to samples shown as red circles in PCA plots) well within south Asian (SAS) populations in principal component 1 and 3 (PC1 and PC3) (Fig. 2a,b), and on the edge of the SAS populations in principal component 2 (PC2) towards the European (EUR) populations (Fig. 2a,b). The localization of both H1 and H2 in PC2 suggests some ancestral link between the Punjabis and European populations. This notion is further strengthened by a recent mitochondrial DNA based analysis where the authors identified clustering of Punjabi population with SAS populations and Eurasian (mixed European and Asian parentage) lineage indicating an ancestral connection between the Punjabi and the EUR populations^16^.

Taken together, we have sequenced whole genomes of two individuals i.e. H1 and H2 from a family of Pakistani descent. We identified 448,544 and 470,683 novel variants in H1 and H2 genome, respectively representing nearly 12% of the total variants identified in each genome. A total of 740,825 variants identified in these genomes were not present in the Caucasian reference genome. PCA based evaluations localized these genomes to SAS populations with an ancestral link to the EUR populations.

## Additional information

**How to cite this article**: Khan, S. Y. *et al*. Whole genome sequencing data for two individuals of Pakistani descent. *Sci. Data* 5:180174 doi: 10.1038/sdata.2018.174 (2018).

**Publisher’s note**: Springer Nature remains neutral with regard to jurisdictional claims in published maps and institutional affiliations.

## Supplementary Material



## Figures and Tables

**Table 1:** Summary of whole genome sequencing data.

Sample ID	Read Length (bp)	Total Reads (10^6^)	Mapped Reads (10^6^)	Mapped reads	Sequenced bases (Gb)	Mean Depth (x)
H1	2×100	760.62	706.49	93%	70.64	22.73 (~23)
H2	2×100	835.45	778.12	93%	77.81	25.10 (~25)
